# Mobile Devices and Sensors for an Educational Multimedia Opera Project

**DOI:** 10.3390/s23094378

**Published:** 2023-04-28

**Authors:** Roger B. Dannenberg, Jorge Sastre, Stefano Scarani, Nuria Lloret, Elizabeth Carrascosa

**Affiliations:** 1 Department of Computer Science, Carnegie Mellon University, Pittsburgh, PA 15213, USA; 2 Institute of Telecommunications and Multimedia Applications, Universitat Politècnica de València, 46022 Valencia, Spain; 3 Department of Sculpture, Universitat Politècnica de València, 46022 Valencia, Spain; 4 Institute of Design and Manufacturing, Universitat Politècnica de València, 46022 Valencia, Spain; 5 Faculty of Education, Universitat de València, 46022 Valencia, Spain

**Keywords:** mobile technologies, sensors, music systems, Soundcool, opera creation

## Abstract

Interactive computer-based music systems form a rich area for the exploration of collaborative systems where sensors play an active role and are important to the design process. The Soundcool system is a collaborative and educational system for sound and music creation as well as multimedia scenographic projects, allowing students to produce and modify sounds and images with sensors, smartphones and tablets in real time. As a real-time collaborative performance system, each performance is a unique creation. In a comprehensive educational project, Soundcool is used to extend the sounds of traditional orchestral instruments and opera singers with electronics. A multidisciplinary international team participates, resulting in different performances of the collaborative multimedia opera *The Mother of Fishes* in countries such as Spain, Romania, Mexico and the USA.

## 1. Introduction

The technological development of the last decades has influenced teaching and learning processes [1]. In the field of music education, technology has significantly transformed the practices of musical creation and composition with the incorporation of new hardware, software and 2.0 tools [2]. The utilization of mobile devices and sensors in education has opened a whole range of possibilities for collaborative work in comprehensive educational projects. In the case of artistic projects, technology allows for the introduction of a variety of multimodal languages that enrich both the creative and educational process and the artistic result [3].

**Figure 1:**
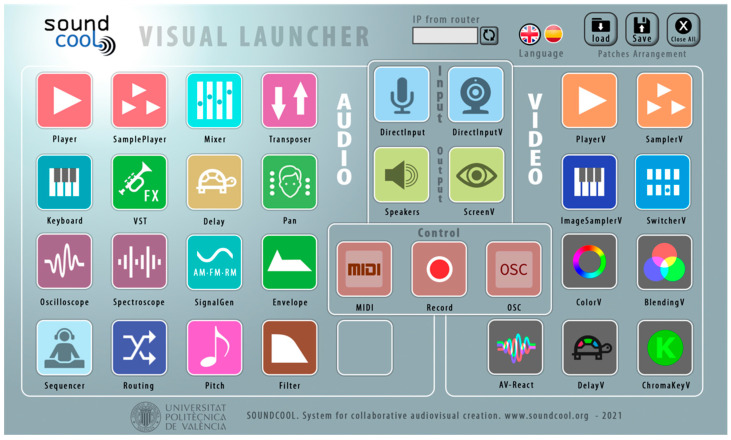
Soundcool for Mac or PC. Audio modules on the left, video modules on the right.

**Figure 2:**
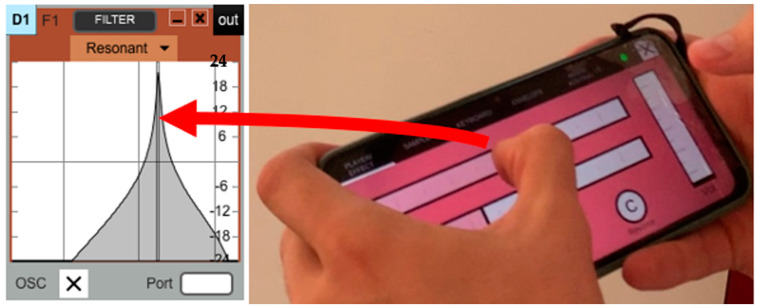
Signal processing parameters such as the frequency of this resonant filter (shown at left) can be controlled over Wi-Fi or Internet using the Soundcool OSC app for smart-phone/tablet (right).

*Soundcool* [4] is an interactive computer-based system for collaborative sound and visual creation using smartphones, tablets and augmented reality, where different kinds of sensors play an active role. Soundcool is developed by the Performing Arts and Technology Group (PerformingARTech) of the Universitat Politécnica de Valencia (Valencia, Spain) with the collaboration of Carnegie Mellon University (Pittsburgh, PA, USA). The system can be downloaded for free at http://soundcool.org (accessed on 24 April 2023) and consists of a set of modules such as microphone or webcam, audio, image and video players and effects, filters, mixing tables, chroma key, video switchers, etc., that work on Mac or PC computers (see Figure 1). Each of these computer modules can be controlled via Wi-Fi or remotely via the Internet by participants using mobile phones or tablets (see Figure 2), or with augmented reality glasses, enabling the collaborative live production of musical, sound and audiovisual projects. For communication, this modular system uses Open Sound Control, designed to share information in real time over a network of media devices.

A multidisciplinary international team of researchers with technical, artistic and pedagogical profiles developed this system in line with the philosophy of STEAM education (Science, Technology, Engineering, Arts and Mathematics) [5] and project-based learning (PBL) [6].

Soundcool is used in different fields, from the artistic professional to the educational, and, more recently, to the health sector as a system used in non-pharmacological therapies in situations of neurodegenerative diseases [7]. However, it is easiest to see the functioning of Soundcool in educational programs and projects originating in 2013, including (1) tests at a secondary school [8], (2) the first educational Soundcool Erasmus+ European Project KA201: “Technology at the service of learning and creativity: weaving European networks through collaborative musical creation,” involving several primary schools, a secondary school and music schools from Italy, Portugal, Spain and Romania [9], (3) the collaborative creation for socially distanced education developed for the pandemic situation [10], and (4) developments for a Soundcool web-based system [11]. See [12] for a history of Soundcool, awards, Soundcool free edX courses, funding and a summary of its educational, professional and functional diversity projects. See https://soundcool.org/publicaciones/ (accessed on 24 April 2023) for technical and educational references on Soundcool. See also the YouTube channel @Soundcoolproject (https://www.youtube.com/c/Soundcoolproject, accessed on 24 April 2023) for video playlists including Soundcool software, tutorials, socially distanced remote projects and professional performances. See also https://bit.ly/2P2CaXB (accessed on 24 April 2023) for educational projects and performances, and see https://bit.ly/39MygwN (accessed on 24 April 2023) for Soundcool educational resources. In 2019, the Soundcool team was invited by the World Science Festival held that year in New York to give a workshop and a performance on its control with augmented reality glasses by dancing [12]. Teachers and students from more than 74 countries have enrolled in the edX Soundcool courses, and the Soundcool team use Soundcool social networks (found on top of the Soundcool webpage) to share related performances and projects. 

Among all of the Soundcool activities, the most emblematic application is the creation of the Opera *La Mare dels Peixos*. The goals of this work were to use Soundcool in a large-scale educational project of creating an opera, to measure its impact, to analyze new possibilities and to improve both the technology itself and the artistic and educational processes. This resulted in different performances of this opera in countries such as Spain and Romania supported by two Educational Erasmus+ European projects, and in America in Mexico and the USA with universities, music schools and high schools. The USA performance was translated into English with the title *The Mother of Fishes*.

In this opera, Soundcool was used to extend the traditional orchestra and opera singers with electronics. It allowed students to create music and sound effects and produce them in real-time during the performance, as well as manipulate sound parameters of live and recorded sound and music through a variety of technological resources (sensors, smartphones and tablets, etc.). The visual and multimedia part of the artistic production was enriched in one of the performances with video mapping. In this project, the role of the students was to participate in all the processes of creating the opera, including sound and visual creation. Because the technology allowed students to produce and modify sounds and images with sensors, smartphones and tablets in real time as they manipulated the different devices, each of these performances generated a unique creation. At the visual level, students participated in the co-creation of multimedia scenography.

At the music level, in addition to offering students an approach to the often elitist genre of opera and to classical, contemporary and electroacoustic music, students were able to participate in the co-creation of the artistic product, as real-time composers, incorporating their musical discourse with that of the composers of the written part of the score (Dannenberg and Sastre). Students, using their own creativity, and informed by their different backgrounds, enriched the artistic product with their interactivity, multimodality and variety of musical ideas. This results in a confluence of artistic, literary, musical, visual and technological discourses in a multimodal context. The multiplicity of interactions between the different interpreters added a multicultural dimension, making the final product of the opera more complex and meaningful.

At the educational level, interdisciplinary work between professionals from different sectors in the fields of telecommunications, computer science, music, arts and design (researchers, programmers, professional singers, musicians and dancers, set designers, make-up artists, composers and conductors) together with students in primary, secondary and university levels allowed for a paradigm shift contributing to the quality of teaching-learning processes through a high-impact educational activity based on STEAM project-based learning (PBL) and Service Learning methodologies. Furthermore, the collaborative creative processes that occurred during every stage of the creation of the opera was cognitively stimulating because the performers put into play higher-order thinking skills that take place in the process of musical interaction and co-creation. Higher-order cognition is defined by Levine [13] as a range of sophisticated thinking skills including concept acquisition, systematic decision making, evaluative thinking, brainstorming, creativity, and rule use.

The new national Spanish curriculum makes explicit reference to the importance of developing audiovisual communication in students and the need for the development of key competences, such as competence in linguistic communication, digital competence and competence in cultural awareness and expression [14]. Children’s and teenagers’ experiences in and out of school settings include a range of modalities, including visual, sound, textual and graphic design elements, and are becoming increasingly more complex, necessitating a rethinking of how multimodal ensembles function across a range of educational and social contexts. Participation in this type of project allows for multimodal literacy of students (especially audiovisual and media literacy) by understanding how different languages and codes work and through the possibility of exploring and manipulating them. Digital competence implies the safe, healthy, sustainable, critical and responsible use of digital technologies for learning, for work and for participation in society, as well as the interaction with them. Digital competency includes information and data literacy, communication and collaboration, media education, digital content creation, security (including digital well-being and cybersecurity-related skills), digital citizenship, privacy, intellectual property, problem solving and computational and critical thinking. Competence in cultural awareness and expression involves understanding and respecting how ideas, opinions, feelings and emotions are creatively expressed and communicated across cultures and through a wide range of artistic and cultural expressions. It also implies a commitment to understanding, developing, and expressing one’s own ideas and sense of place or role in society. It also requires an understanding of one’s evolving identity and cultural heritage in a world characterized by diversity, as well as an awareness that art and other cultural manifestations can be a way of looking at the world and giving it shape.

## 2. Related Work

Opera creation has been used in project-based education for many years. The Metropolitan Opera Guild has offered the Creating Original Opera program in which school students form an opera company and produce an original opera [15], adapted in Spain into a program called “LÓVA” [16], where Soundcool has also been used. The central goal is to learn about art, but the process reinforces skills of reading, writing and math as well as social and collaborative skills. In a similar vein, many have promoted STEAM (Science, Technology, Engineering, Arts, and Math) [5], as opposed to STEM, as a more comprehensive and relevant approach to curriculum design for a world where “soft” skills of creativity, collaboration, persuasion are as important as “hard” skills of math and science.

Computer Music in particular has formed the basis for STEAM practice in several projects. EarSketch is a widely used system that introduces programming by having students write programs to construct and edit music using professionally produced audio “loops” as source material [17]. BlockyTalky is a music system construction set for young students that features a simple block-based visual programming language for small programmable music devices [18]. These devices synthesize sound, receive sensor input and communicate via messages. Students configure microcontroller devices, sensors and software to create and perform interactive musical systems of their own design. The Soundcool project places more emphasis on music education and creation, but it shares the goals of EarSketch and BlockyTalky to promote computer literacy, collaboration and creativity through project-based learning.

Apart from The Metropolitan Opera Guild, some other opera educational initiatives are the Los Angeles Opera which supports educators, helping to integrate opera into the classroom and provide an opportunity to participate in the creation process [19]; Royal Opera House Youth Opera programs provide children aged 7 to 13 with rigorous music and drama training, creative projects and the chance to perform in world-leading opera productions with the Royal Opera [20]; Youth Opera of the Welsh National Opera is an award-winning training program for young people, aged from 8 to 25 years, who love to sing and perform [21]. The majority of these initiatives are based on students participating in an opera and/or the creation of an opera. However, none of them include technology. On the other hand, the main objective of “TRACTION Opera co-creation for a social transformation” is developing new technologies to establish an effective participatory production workflow and to explore novel audio-visual art representation formats [22]. TRACTION invites the participation of diverse communities, not only young people and students. The project includes a Co-Creation Space, which is a web-based platform that acts as a media repository and collaborative tool for uploads, visualization and communication around media objects; a Co-Creation Stage, which is a web-based tool that supports both live and on-demand media to enable art professionals and individuals from the TRACTION’s targeted communities to remotely participate in engaging and immersive opera shows, for instance singing remotely; an Immersive Adaptive web-based 360° opera video player and a Social Virtual Reality tool for participants’ communications after attending a VR show. However, none of this includes the participation of the students in the creation and performance of the opera with technology.

In our project, we work with Soundcool, which has been developed specifically for young students and collaborative classroom use, particularly through the use of mobile phones and tablets, which extend interactive control to an entire classroom through the technologies of Wi-Fi or the Internet for remote connections, and intuitive touchscreen sensors and other interfaces. Many other systems exist, including SuperCollider [23], offering a real-time programming language for constructing music systems, Max MSP [24], a very popular music and media system based on a visual programming language, and Ableton Live [25], a digital audio workstation (DAW) that is particularly designed for use in interactive live performance. All of these can be controlled remotely and allow real-time sensor input, but none are specifically designed for young students or collaborative use. Soundcool aims for a “sweet spot” between the generality offered by programming languages and the ease of use of single-purpose applications [12].

## 3. Soundcool in Opera Productions

The multimedia opera has been premiered at the Opera House “Palau de les Arts Reina Sofia” in Valencia (Spain) by students from two music schools, a primary school and a high school, in December 2016. The same year, its Act I was also performed with secondary level students of an art program at the theater of the Liceul de Arta Baia Mare (Romania), supported by the Erasmus+ European project. In 2017, Act I was also performed at the Monterrey Institute of Technology and Higher Education (ITESM) Puebla Campus (Mexico) by university students, and at the Theater of the Musical Society of Canet d’en Berenguer (Valencia, Spain) and at the Auditorium of Torrent (Valencia, Spain) by music students. In 2018, the full opera was performed at the ITESM Puebla Campus (Mexico) by university students, and at La Rambleta Theater of Valencia (Spain) with children from a cultural association. In May 2019, it was performed again at the Valencian Opera House “Palau de les Arts Reina Sofia” (Spain) by primary school students. Afterwards, it was performed at the Universitat Politècnica de València (Valencia, Spain) by primary school students in 2019 inside another educational Erasmus+ European project. Finally, it was performed at the National Center for the Arts (CENART) in Mexico City (Mexico) with a co-production of the Monterrey Institute of Technology and Higher Education and CENART in November 2019, by university students and younger music students, and in February 2020 at the CAPA Theater in Pittsburgh (USA), with high school and university students and professionals (see the opera project webpage at https://soundcool.org/en/opera/, accessed on 24 April 2023, teaser at https://youtu.be/iREw70OcvmE, accessed on 24 April 2023, and additional videos at https://www.youtube.com/@Soundcoolproject/playlists?view=50&sort=dd&shelf_id=5, accessed on 24 April 2023).

Each project and performance has been adjusted to the available resources, and performances continue to evolve with the integration of new technological elements. This article considers the project carried out in Pittsburgh.

## 4. Collaborative Control Using Sensors in Interactive Systems

The use of interactive systems based on sensors or remote control is certainly not new. Today, most audio applications allow remote management of their mixing systems and control of many functions via a phone or tablet. However, Soundcool offers something that is still practically unique: the distributed control of functions, allowing not just one user, but a group of users, to control the same system simultaneously, distributing the different modules, or the different functions of generation, management, and transformation of audiovisual material. This characteristic is at the center of group dynamics typical of a class where the fundamental point is cooperation within the group to create the desired performance, much as an orchestra cooperates to perform a symphony. Cooperative control by the group is also an innovative language in the field of art itself, allowing a type of collaboration that only partially exists within the group performance experience of bands, choirs, and orchestras. In typical musical practice, each interpreter contributes to the whole through the sole control of a single instrument. In Soundcool, electronic instruments are created with control parameters that are delegated across several performers, analogous to an instrument where one person presses the strings, another pinches or rubs them, and yet another modifies their dynamics, etc. In practice, collaboration in Soundcool is both in parallel, as normally happens in instrumental groups, and in a serial form where single sounds are subject to a series of modifications. This kind of cross-collaboration implies that the people involved must pay close attention to every small contribution in order to achieve the desired balance.

**Figure 3:**
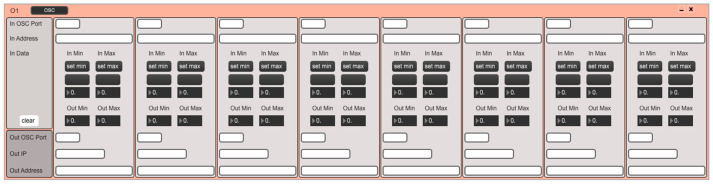
Soundcool OSC module for redirecting incoming OSC commands to any active module in the application.

Soundcool uses the Open Sound Control communication protocol [26] for wireless communication with control devices to facilitate their integration into collaborative performances. OSC is one of the most-used protocols for this kind of communication and in fact, Soundcool, in addition to being controlled by the original iOS and Android applications created specifically for Soundcool, is open to control by any other system that uses the same protocol and correct syntax. This means that Soundcool can immediately be controlled by custom-built sensors, programming languages such as Max [24] or PureData [27], or generic control applications such as TouchOSC [28], which leverage touch sensors and accelerometers in mobile devices. In short, the step between a sensor system and Soundcool is short; it is sufficient to translate the incoming data from any sensor into variable values and send them via OSC to Soundcool modules. This allows for both creative design and for the flexible interconnection of multiple devices, enabling collaborative sound control. The possibilities for creating personal and group control systems are open to the imagination. To facilitate this open control even more, a module has been developed in Soundcool that redirects any incoming OSC command to any active module in the application (see Figure 3).

**Figure 4:**
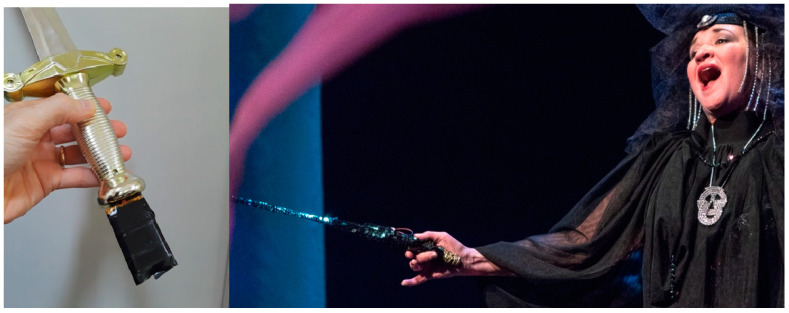
The sword prop and “magic wand” used in the opera production. Each has a 9 DOF sensor and microcontroller with Wi-Fi to communicate with a computer running Soundcool. Performers’ gestures trigger and control sound effects in real time.

The open-ended control possibilities of Soundcool are an alternative to offering an internal programming language as seen in other systems such as Supercollider and Max. Basically, Soundcool can be seen as a component whose capabilities can be extended through communication, delegating control to another component, which allows a choice of programming language. A concrete example can be found in the version of the work *The Mother of Fishes*, made in Pittsburgh, where a sword and magic wand (stage props) enclosed motion sensors used to control the generation and modification of sounds in Soundcool. Sensors were 9-degree-of-freedom inertial measurement units interfaced to ESP-32 microcontrollers with integrated Wi-Fi transceivers. The resulting sensors, including batteries for power, were about 6 × 3 × 2 cm, and were easily added to the handles of a sword prop and a “magic” wand (see Figure 4).

For both sensors, the data is processed to derive useful control signals. The sword, when swung, produces a “whooshing” sound by modulating the center frequency of a bandpass filter (similar to the one shown in Figure 2) with noise input. The main signal feature is the overall RMS of the 3D accelerometer signal. Additional limiting and scaling are used to derive the desired filter center frequency, which is transmitted to Soundcool. (See video of sword whooshing gesture and sound: https://youtu.be/tBcnFzCKi3A?t=44, accessed on 24 April 2023). On the other hand, the wand is used to fill the stage with echoes of evil laughter. When the witch character presses a hidden button on the wand, a recording of her laughter is injected into a network of feedback delay units in Soundcool. The audio levels and degree of feedback are controlled to intensify the effect as the wand is raised, as if evil laughter is emitting from the wand itself, and the effect diminishes as the wand is lowered. Again, the accelerometer data is processed, in this case by combining two axes of acceleration (due to gravity) to estimate the overall tilt of the wand and convert this to gain control in Soundcool (See video of the wand evil laughter effect: https://youtu.be/tBcnFzCKi3A?t=131, accessed on 24 April 2023). The mapping of sensors to Soundcool controls could be done directly within the sensor microcontrollers, but for ease of development, we perform all the signal mapping using a scripting language in the same laptop computer that runs Soundcool. Conditioning signals and mapping them to specific sound synthesis parameters can be implemented in a variety of ways, as long as the end result is an OSC message to Soundcool. Moving this computation outside of Soundcool is seen as a feature rather than a limitation because it keeps the design simple and supports many implementation tools and strategies.

**Figure 5:**
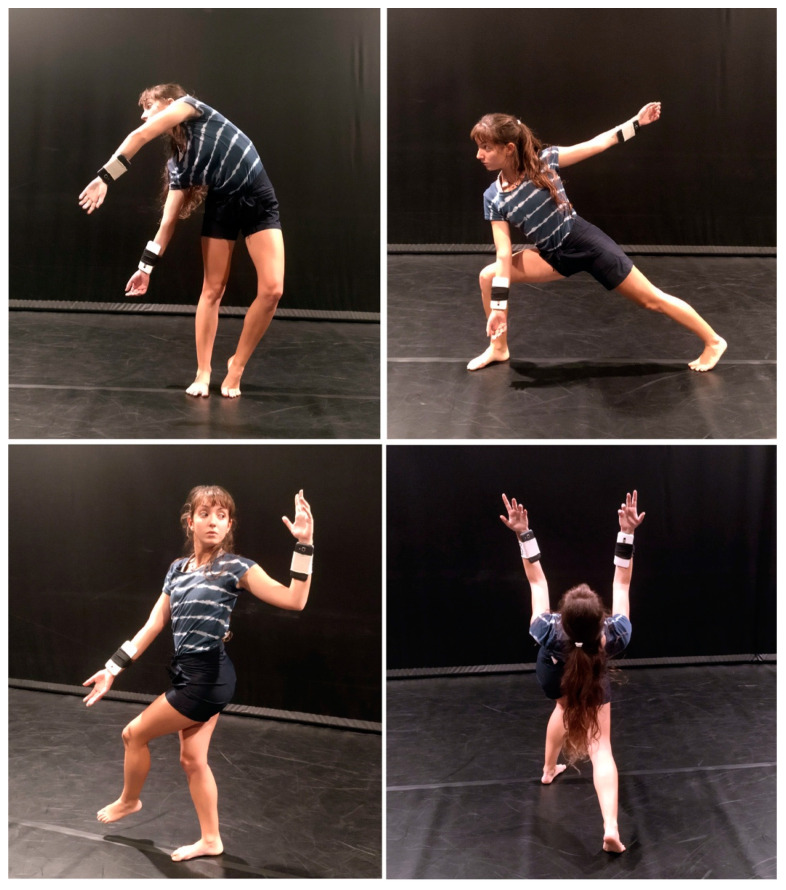
Experiments using recycled smartphone as a sensor for dance. (dancer: Noelia Sánchez Gómez, ph. Laura Basterra Aparicio).

In a similar manner, we have recently worked on the use of recycled smartphones [29] (Figure 5), or older devices no longer suitable for everyday use, but still fully functional as sensing devices. In this case, the data produced by internal sensors (inclination, acceleration) are sent to Soundcool through the OSChook Android application [30] and recycled Wi-Fi hubs, and finally used to control the generation of sound environments responding to choreographic gestures in dance.

The sensor data can be sent directly to Soundcool’s OSC module, or we can implement more complex processing with additional software. In this case, the data is received by a patch programmed in Max [24], mapped to an appropriate range of values for a Soundcool module, and then sent to the module within the Max process. This approach of programming additional functions is also used to create module prototypes that will later be released as new modules in Soundcool.

## 5. Outcomes from Opera Productions

An analysis of educational practices with Soundcool was carried out within the European Erasmus+ Project KA201 (2017) using a mixed design consisting of questionnaires, direct observation and performance analysis in four educational institutions with a total of 32 students and four teachers. The data collection methodology included an initial evaluation before the start of the project and a final evaluation. The results of the analysis indicated that the competency levels of participating students increased significantly. These results were consistent regardless of student age, gender, country of origin and background [31].

The English version of the complete opera was held in the USA in February 2020. In this performance, the students of the Creative and Performing Arts Magnet School (CAPA) in Pittsburgh (USA) carried out all the sound creation of the opera, mostly using Audacity (http://bit.ly/tmof-audacity, accessed on 24 April 2023), and performed the electronic parts live. The students were taught sound design, digital audio editing, and the use of Soundcool by one of the co-authors (Dannenberg) in order to create and perform sound effects for the opera. Although most of the sounds can be described as “sound effects,” it should be noted that many electronic sounds are specifically called for in the orchestral score and are considered to be part of the orchestration. The details of these sounds are deliberately left open to interpretation and the sound design has been realized by different student groups in multiple productions of the opera. Thus, students are responsible for taking an active creative role in each opera production.

For the 2020 production, the students also realized a new electronic overture for the opera. The overture utilizes audio time stretching, found sounds, panning, pitch shift and volume control, both in the composition and the live performance. The 2020 performance utilized four students performing live using mobile phones as controllers as well as the magic sword and witch’s wand sensors and effects described above. Examples of the sensors and the electronic overture, as well as the complete opera and additional documentation, can be seen online [32,33,34]. See also the summary and photos (https://soundcool.org/en/operas/the-mother-of-fishes-teatro-capa-eeuu-2020/, accessed on 24 April 2023).

At the technical level, this comprehensive project contributed to the evolution of technology. The team of researchers, technicians and programmers worked to meet the needs of the users that arose in the practical application of Soundcool, resulting in its continuous improvement.

Regarding social impact, the project has involved the direct participation of more than 800 students, teachers, and professionals from countries such as Romania, Spain, Mexico, UK, USA, etc. Overall, more than 500 students from primary schools, high schools, universities, music schools, music conservatories, dance conservatories, art schools, etc., have participated, and the project has been a finalist at the Spanish Creative Cup. See for instance the work with Spanish primary school students (https://youtu.be/2y5anpWTx-g, accessed on 24 April 2023) or with Mexican university and young music students (https://youtu.be/5ym2d3JroZA, accessed on 24 April 2023). 

## 6. Discussion

A fundamental design element of Soundcool is the control of sound through everyday devices such as smartphones and tablets. The possibility of treating these devices as control surfaces introduces the possibility of fast and multitouch control. It also enables the essential ingredient of interaction, even remote interaction, between people that characterizes Soundcool [10]. Different people cooperate in real time to control a chain of sound and image generation and manipulation processes. This enablement of close real-time collaboration in groups is a rare element in the world of new technologies.

The use of the OSC protocol is an important feature that opens Soundcool to extension through the use of added sensors and external programs ranging from short control scripts to substantial software systems. This is illustrated by the example of added sensors for *The Mother of Fishes* performance and the use of smart phones as sensors for dancers. Ultimately, it is always a question of translating sensor data to the common language or protocol of OSC, which can then be used to control Soundcool modules and create an interactive sound composition or environment.

We are actively pursuing off-the-shelf sensors such as Leapmotion and UltraLeap [35], augmented reality systems such as Microsoft Hololens already in use with Soundcool [12] (pp. 82–83]) [36] and Oculus [37], body recognition systems such as Kinect (used with Soundcool but no longer available) and PoseHook [38]. In addition to building modular systems around Soundcool as the “hub,” we see great promise in expanding the idea of modular systems to include many other advanced software systems used in performance, music and theater. The use of OSC over sockets and Wi-Fi offers an effective interconnect with visual programming languages such as Max and PureData, scripting languages such as Python, and many other applications, which further expand the possibilities for sensors and collaborative control in artistic productions.

Because Soundcool is free and supported by many educational materials, we know it is used in many different countries independently of the authors’ projects. Many people have enrolled to the edX Soundcool courses without any direct involvement with the Soundcool team. In addition, another Educational Erasmus+ European project using Soundcool is planned, and we are participating on the Teaching Innovation Project “Learning and Innovation with Sound” with the Education Faculty of the University of Valladolid (Spain) and also in Red Planea (https://redplanea.org/recursos/soundcool-2/, accessed on 24 April 2023), a resource for the Spanish network for art and education.

Success with the opera has led to a new project, the opera *Felicità* on the theme of bullying and harassment. The opera, involving the authors, consists of different bullying and harassment situations based on an original idea from Lloret, with libretto by Idoia Uribarri and Lloret, instrumental music and Soundcool live processing by Sastre and Dannenberg and interactive video projections by Scarani. The work includes contemporary and street dance to connect more easily with students. Each act deals with a different kind of bullying or harassment and can be performed independently. Though a theatrical version was premiered at the Monterrey Institute of Technology and Higher Education (Mexico) in 2021, the first opera version was premiered on October 2022 in Spain with an audience of 800 students. We plan to add more acts to deal with more situations as the project evolves.

Considering the future of Soundcool, we are developing new modules such as a video-mapping module, we continue to develop a web-based Soundcool implementation [11], and we will further explore the reuse of sensors from older mobile devices as a low-cost and available technology. We also expect to increase our research and practice using Soundcool in the area of neurodegenerative diseases, Attention Deficit and Hyperactivity Disorder (ADHD) and other conditions.

## 7. Conclusions

Soundcool is an easy and intuitive system to use, where simple modules hide the complexity of the underlying signal processing that includes both sophisticated audio processing, such as real-time pitch shifting, and video processing such as compositing. An important feature of Soundcool is its interconnection with external devices and software. It uses mobile devices with touch screens as sensors to enable groups to collaborate in live performance. We also develop microcontrollers with Wi-Fi to connect custom sensors such as accelerometers to capture motion for sound and video control.

These possibilities are illustrated by the opera *The Mother of Fishes* and our example with dancers and accelerometers. The modular “construction kit” organization of Soundcool allows for the rapid creation of interactive sound systems controlled by groups of performers using a variety of sensors ranging from mobile device touch screens to custom-built devices. These systems have enabled both students and professionals to creatively explore interactive sound design and music composition.

The results from the Erasmus+ European project named above [31] and other educational projects carried out since the first Soundcool version was released almost ten years ago [12] encourage us to think that Soundcool has excellent potential to promote creativity, collaborative work, interdisciplinarity and cognitive development in different areas and across different cultural systems. Due to the versatility and flexibility of the system, the ease of use of the interfaces and the low cost of the equipment, Soundcool has applications beyond artistic and educational contexts. Soundcool and the collaborative co-creation it supports are easily generalizable to other contexts such as the field of health and well-being, therapeutic use with patients with functional diversity, students with special needs, geriatric patients and Alzheimer’s patients among others. The Soundcool project continues to promote collaboration and international development.

## Data Availability

Not applicable.
